# Transient and intensive pharmacological immunosuppression fails to improve AAV-based liver gene transfer in non-human primates

**DOI:** 10.1186/1479-5876-10-122

**Published:** 2012-06-15

**Authors:** Carmen Unzu, Sandra Hervás-Stubbs, Ana Sampedro, Itsaso Mauleón, Uxua Mancheño, Carlos Alfaro, Rafael Enríquez de Salamanca, Alberto Benito, Stuart G Beattie, Harald Petry, Jesús Prieto, Ignacio Melero, Antonio Fontanellas

**Affiliations:** 1 Gene Therapy and Hepatology Area, Centre for Applied Medical Research (CIMA), University of Navarra, Navarra, Spain; 2 Research Center, Hospital Universitario 12 de Octubre, Madrid, Spain; 3 Department of Radiology, Clínica Universitaria de Navarra, University of Navarra, Pamplona, Spain; 4 Research and Development, Amsterdam Molecular Therapeutics (AMT), Amsterdam, The Netherlands; 5 CIBERehd, Barcelona, Spain; 6 Division of Gene Therapy and Hepatology, CIMA and CUN, Universidad de Navarra, Avda. Pio XII, 55, Pamplona, 31008, Spain

**Keywords:** Adeno-associated virus serotype 5, Neutralizing antibodies, Re-administration, Vector Immunology/Host Responses, Immunomodulation

## Abstract

****Background**:**

Adeno-associated vectors (rAAV) have been used to attain long-term liver gene expression. In humans, the cellular immune response poses a serious obstacle for transgene persistence while neutralizing humoral immunity curtails re-administration. Porphobilinogen deaminase (PBGD) haploinsufficiency (acute intermittent porphyria) benefits from liver gene transfer in mouse models and clinical trials are about to begin. In this work, we sought to study in non-human primates the feasibility of repeated gene-transfer with intravenous administration of rAAV5 vectors under the effects of an intensive immunosuppressive regimen and to analyze its ability to circumvent T-cell immunity and thereby prolong transgene expression.

****Methods**:**

Three female *Macaca fascicularis* were intravenously injected with 1x10^13^ genome copies/kg of rAAV5 encoding the human PBGD. Mycophenolate mofetil (MMF), anti-thymocyte immunoglobulin, methylprednisolone, tacrolimus and rituximab were given in combination during 12 weeks to block T- and B-cell mediated adaptive immune responses in two macaques. Immunodeficient and immunocompetent mice were intravenously injected with 5x10^12^ genome copies/kg of rAAV5-encoding luciferase protein. Forty days later MMF, tacrolimus and rituximab were daily administrated to ascertain whether the immunosuppressants or their metabolites could interfere with transgene expression.

****Results**:**

Macaques given a rAAV5 vector encoding human PBGD developed cellular and humoral immunity against viral capsids but not towards the transgene. Anti-AAV humoral responses were attenuated during 12 weeks but intensely rebounded following cessation of the immunosuppressants. Accordingly, subsequent gene transfer with a rAAV5 vector encoding green fluorescent protein was impossible. One macaque showed enhanced PBGD expression 25 weeks after rAAV5-*pbgd* administration but overexpression had not been detected while the animal was under immunosuppression. As a potential explanation, MMF decreases transgene expression in mouse livers that had been successfully transduced by a rAAV5 several weeks before MMF onset. Such a silencing effect was independent of AAV complementary strand synthesis and requires an adaptive immune system.

****Conclusions**:**

These results indicate that our transient and intensive pharmacological immunosuppression fails to improve AAV5-based liver gene transfer in non-human primates. The reasons include an incomplete restraint of humoral immune responses to viral capsids that interfere with repeated gene transfer in addition to an intriguing MMF-dependent drug-mediated interference with liver transgene expression.

## **Background**

Acute intermittent porphyria (AIP) is an autosomal dominant inherited disease clinically characterized by life-threatening acute neurologic attacks and is biochemically defined by partial deficiency of porphobilinogen deaminase (PBGD) activity in the liver. Recombinant adeno-associated viral (rAAV) vectors are a promising gene therapy tool for the correction of genetic disorders [1-4]. Recently, we and others [5,6] have reported sustained hepatic expression of human PBGD in the liver of a mouse model of AIP transduced with rAAV-*PBGD* vectors. A multi-center national phase I/II clinical trial with rAAV vector serotype 5 will be executed as a prophylactic treatment for patients with a severe status of AIP.

The genome of the rAAV vector persists predominantly in episomal forms, thereby increasing safety by reducing the risk of insertional mutagenesis. However, this feature may favour loss of genomes and transgene expression over time due to hepatocyte turn-over. Although the hepatocyte proliferation rate is normally very low in adult mammals, sustained transgene expression throughout the life of the patient will require repetitive administration. However, rAAV-mediated liver gene transduction may be compromised by preexisting immunity to AAV. First, neutralizing antibodies (nABs) at the time of administration directly hamper the clinical DNA delivery system [7]. Secondly, prior infection of the patients with natural or recombinant AAVs leads to formation of memory CD8+ T cells which readily undergo activation upon re-exposure to the AAV capsids [8,9].

Delivery of viral vectors under transient pharmacological immunosuppression is a conceivable strategy to diminish host immune responses against vector capsid proteins and may provide a strategy to permit repeated rAAV vector administrations [10-13]. However, laboratory mice did not reproduce T cell–mediated destruction of rAAV-transduced hepatocytes [14]. As a consequence, experiments in primates are clearly necessary to guide clinical development because mouse experiments are not considered predictive from the point of view of immunogenicity.

Recently, we have demonstrated in non-human primates that the adaptive immune response to a first generation adenoviral vector could be averted by a course of immunosuppression which combines B-cell depletion and T-cell inhibition with clinically available drugs [15]. Such a regimen permitted up to four re-administration cycles. The aim of this study was to investigate in non-human primates the feasibility of gene re-transfer with intravenous administration of rAAV5 vectors under the effects of the same intensive immunosuppressive (IS) regimen used with the first generation adenoviral vector.

Results from the hemophilia trials [8,9] that involved serotype 2-based rAAV vectors have demonstrated that the activation of capsid-specific cytotoxic CD8+ T cells extinguishes transgene expression in a matter of weeks. In a more recent clinical trial using serotype 8 self-complementary rAAV vector, factor IX transgene expression was preserved in two patients when a short course of glucocorticoid therapy was given [16]. Thus, the second aim of this study was to investigate the ability of transient IS regimes to circumvent T-cell immunity and thereby prolong transgene expression.

## **Methods**

### **Construction of plasmids and production of rAAV5**

The expression cassette encodes the human cDNA of the housekeeping PBGD isoform or the reporter genes (luciferase or enhanced green fluorescence protein, eGFP) flanked by the inverted terminal repeats from AAV2. Transgenes are under the control of the liver specific human α-1-antitrypsin promoter with regulatory sequences from the human albumin enhancer (EalbAAT promoter) and include the human PBGD polyadenylation sequence [5] (bases 9550–9655 GenBank Accession No. M95623). The rAAV5 vectors were generated in Sf9 insect cells [17]. Vector particles were purified and final concentration of different batches was determined by quantitative polymerase chain reaction (QPCR), as previously described [5].

### **Macaque experiments and immunosuppression treatment**

**Figure 1:**
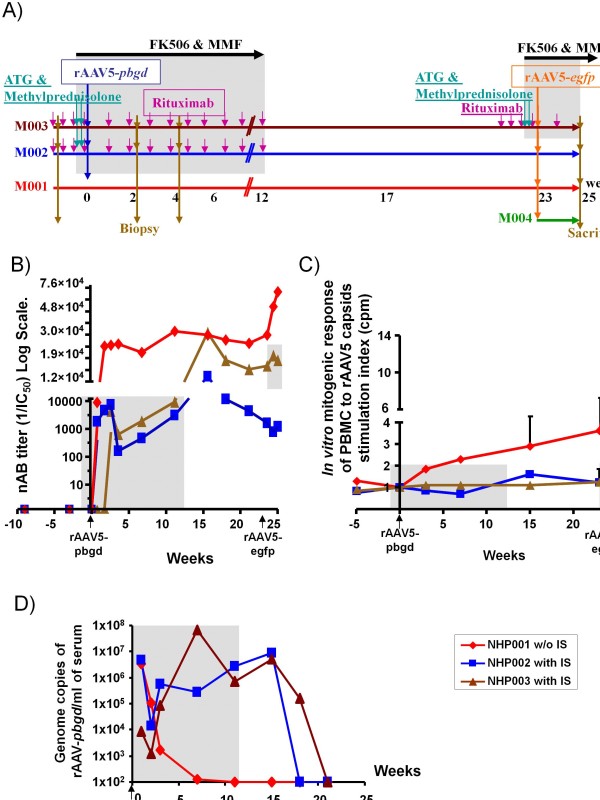
**An immunosuppressive pharmacological regimen delays and weakens immunity against rAAV5 capsid antigens. A)** Schematic timeline representation of the administration of two rAAV serotype 5 vectors in macaques under combined pharmacological immunosuppression. rAAV5-*PBGD* was injected first and rAAV5-*egfp* second. Animals are colour coded, identified by the NHP-digit code and their timeline represented by a horizontal arrow. **B)** Sequential follow-up of serum rAAV5 neutralizing antibodies in the serum of the colour-coded animals. **C)***In vitro* mitogenic response of peripheral blood mononuclear cells (PBMC) at the indicated time points to rAAV5 capsids, measured by ^3^ H-Thy incorporation. **D)** Sequential follow-up of proviral rAAV5-*PBGD* DNA detection from serum samples of the colour-coded animals by quantitative PCR of vector DNA. Each dot represents the average of two independent measurements for each sample at a time point.

Three female captive bred NHP (*Macaca fascicularis*, 4 to 5-years of age) were intravenously (*i.v.*) injected with 1x10^13^ gc/kg rAAV5-*PBGD* following the schematic time line presented in Figure 1A. A second vector infusion of 3x10^12^ gc/kg rAAV5-eGFP was performed in these three macaques and an additional control naïve macaque. Liver biopsies were obtained one week before and 2 and 4 weeks after the rAAV5-*PBGD* injection. Vector copy number and PBGD activity were assessed from a mix of 4 liver samples corresponding to each animal at each time point.

Macaques were sacrificed twenty five weeks after rAAV5-*PBGD i.v.* injection, corresponding to two weeks after the administration of rAAV5-*egfp*. The control naïve macaque injected with rAAV5-*egfp* was sacrificed two weeks after vector injection.

The B and T-cell IS regimen was administered as described: (i) B-cell-depleting Rituximab (20 mg/kg/dose *i.v.,* Mabthera, ROCHE, Switzerland) at days −9, -6, -3, immediately before rAAV injections and weekly after the viral administration, (ii) two *i.v.* doses of 3 mg/kg of T-cell-depleting anti-thymocyte gamma-globulin (ATG) (Genzyme Polyclonals, SAS, France) at days −2 and −1 before rAAV injection. (iii) Methylprednisolone (Solu-moderin, Pfizer SA, Spain) applied intramuscularly 10 min before the ATG infusion at a dose of 100 mg on day −2 and 50 mg on day −1. (iv) Mycophenolate mofetil (MMF) (CellCept, Roche Pharma AG, Denmark) at a dose of 25–30 mg/kg/day and (v) 0.25 mg /kg/day of tacrolimus (FK506)(Astellas Pharma, Madrid, Spain). MMF and FK506 were given orally from day −2 daily to the end of IS protocol (shaded areas). This protocol maintained serum levels within the range of 2 to 4 μg/mL for mycophenolic acid (the active metabolite of MMF) and 2 to 8 ng/mL for tacrolimus, as measured in a different cohort of *Macaca fascicularis*. Toxicities for all of these agents are well-described in non-human primates.

All animal experiments were approved by the ethics committee of the University of Navarra in accordance with Spanish regulations (study approval CEEA038-09). Experiments in primates are clearly necessary to guide clinical development because mouse experiments are not considered predictive from the point of view of immunogenicity. However, ethical and regulatory constraints limit the number of NHP available for this experimentation to a minimum.

### **Mouse experiments**

The effect of IS drugs on transgene expression was monitored in eight female BALB/c mice intravenously injected with a dose of rAAV5-luciferase (5x10^12^ gc/kg). At day forty, five animals received daily intraperitoneal administration of 30 mg MMF /kg/day during the first week and a combination of MMF, FK506 (0.25 mg/kg/day) and Rituximab (20 mg/kg/day) during the second week. Eighteen BALB/c and nineteen Rag^−/−^ female mice were injected, *i.v.*, with 5x10^12^ gc/kg of rAAV5-*luciferase*. Rag^−/−^ mice have small lymphoid organs and do not contain mature B and T lymphocytes as Rag^−/−^ is needed for the V(D)J recombination of immunoglobulin and T cell receptor genes. Four weeks after vector injection animals were injected *i.p.* with a dose of 30 mg of MMF/kg/day for two weeks. Non-invasive luciferase expression measurements were performed in living mice, as previously described [18].

### **Transgene expression**

Human PBGD transgene expression was measured by enzymatic activity and immunohistochemistry, as previously reported for PBGD [5], or real-time QPCR analysis. Total RNA was purified from homogenized tissue samples using TRIzol® reagent (Invitrogen, Carlsbad, CA). Reverse transcriptions with random primers were performed at 37 °C for 50 min using M-MLV Reverse transcriptase (Invitrogen). Real-time QPCR was performed using primers designed to amplify a 51 base pair fragment of the human recombinant 3´UTR-poly A *PBGD* region (pAPBGDfw5´-GCTAGCCTTTGAATGTAACCA-3´, pAPBGDrv5´-CCTTCAGAACTGGTTTATTAGTAGG-3´), or a 43 base pair region of the eGFP sequence (eGFPfw, 5'–GTCCGCCCTGAGCAAAGA–3'; eGFPrv, 5'–TCCAGCAGGACCATGTGATC–3').. Endogenous mouse *β-actin* sequence (mBactinfw, 5'–CGCGTCCACCCGCGAG–3'; mBactinrv, 5'–CCTGGTGCCTAGGGCG–3'; product length: 193 bp) or endogenous simian β-actin (sBactinfw, 5'–GAGCGGGAAATCGTGCGTGACATT–3'; sBactinrv, 5'–GAAGGTAGTTTCGTGGATGCC–3'; product length: 210 bp) were also amplified to equalize quantity. QPCR was performed in an iQ5 real-time PCR detection system (Bio-Rad, Hercules, CA) using iQ SYBR green supermix. PCR amplification conditions were as follows: 95 °C for 5 min, 40 cycles of 95 °C for 15 sec, 60 °C for 15 sec, 72 °C for 25 s and 80 °C for 10 s; and a final extension for 10 min at 72 °C). A melting curve was generated by raising the incubation temperature from 65 °C to 95 °C to confirm amplification specificity. The amount of each transcript was expressed according to the formula 2^Ct(GAPDH)-Ct(gene)^, where Ct is the cycle at which the fluorescence rises appreciably above background fluorescence.

### **Proviral DNA quantification in liver samples and serum vector detection**

Detection of the specific rAAV5-*PBGD* or rAAV5-*luciferase* plasmid sequences was performed in genomic DNA of harvested samples isolated using the QIAmp Blood&Tissue DNA Mini-kit (Qiagen). Genomic DNA was subjected to QPCR using specific primers as described above for transgene expression. For normalization of amount of genomic DNA, specific primers for murine and simian *gapdh* sequence were used (mGAPDHfw, 5'–CCAAGGTCATCCATGACAAC–3'; mGAPDHrv, 5'–TGTCATACCAGGAAATGAGC–3'; sGAPDHfw 5'–GTCAGTGGTGGACCTGACCT–3'; sGAPDHrv, 5'–TGCTGTAGCCAAATTCGTTG–3').

### **Measurement of humoral and cellular anti-AAV5 responses**

Sera were obtained by tail vein puncture and stored at −80 °C. Neutralizing antibody assays to rAAV5-luciferase were performed with serial dilutions of serum that were mixed with 1x10^9^ genome copies of rAAV5-luciferase. The mix was incubated at 37 °C for 1 h and then the mixture was added to PLC-PRF5 cells (1x10^4^ cells per well) in a 96-well plate. Cells were harvested 144 h later and assayed for luciferase activity (D-luciferin Luciferase kit, Promega) using the Living Image 2.20 software package (Xenogen). Sera were scored as positive for neutralizing anti-rAAV5 antibodies if the light intensity was less than 50 % of that observed when rAAV5 was pre-incubated with negative control sera. Data are reported using a curve adjusted to extrapolate the serum dilution for 50 % inhibition of rAA5-luciferase transduction (IC_50_).

Antibodies directed to viral capsids were titrated by ELISA. Microtiter wells were coated by overnight incubation at 4 °C with purified viral capsids (1 μg/ml) in 0.1 M sodium carbonate buffer. After washing and blocking the wells with Phosphate Buffer Saline and 10 % bovine serum albumin, sera were tested at serial dilutions. Following extensive washing specific binding of Goat anti-monkey IgG, IgM, IgA biotin conjugated (1/10.000 dilution, SAB 1062, Open Biosystem) was developed with streptavidin-avidin peroxidase at 1/250 and TBM substrate (BD biosciences). Antibody titers correspond to the highest serum dilution to yield three times the absorbance of a negative serum and were expressed as the reciprocal value of the dilution.

To evaluate the cellular immune response induced against the vector, monkey leukocytes were purified from peripheral blood by centrifugation through Ficoll-Hypaque (GE Healthcare, Piscataway, NJ) and counted in a Z2 Coulter Counter (Beckman Coulter). *In vitro* mitogenic responses to AAV capsids was measured by [methyl-^3^H] Thymidine incorporation of isolated leukocytes (5x10^5^ cells/mL) cultured for 72 h in medium alone [X-vivo medium (BioWhittaker) supplemented with 2 mM glutamax (Invitrogen), and 1 % penicillin/streptomycin (Invitrogen)] or containing serial dilutions of rAAV5 vector from 10–0.01 μg prot./mL. To assess the ability of cells to respond to mitogens, a mix of the phorbol ester 12-myristate- 13-acetate (0.01 μg/mL) and Ionomicine (1 μg/mL) was used in each experiment (Sigma-Aldrich, St. Louis, MO) as a positive control. [3 H]-thymidine uptake was assessed by filtration on an automatic cell harvester and by measuring nuclear radioactivity (filters) on a scintillation plate reader (Topcount). The stimulation Index (S.I.) was defined as the mean counts per minute (cpm) of the response of the antigen-stimulated cells divided by the mean cpm of the of cell cultures without antigen.

### **Humoral anti-human PBGD protein detection**

The samples were analyzed for antibodies to human PBGD protein by Western blotting. Purified recombinant human PBGD protein was resolved by electrophoresis by 12 % SDS-PAGE and transferred onto a PVDF membrane (Amersham Hybond-PTM, Bucks, UK). After blocking, the membranes were incubated with a rabbit polyclonal anti-PBGD (1:300 H-300, Santa Cruz, CA) as a positive control, or sera from macaques NHP001, NHP002 or NHP003 (diluted 1:3 in saline). After additional washes, the membranes were incubated with an anti-rabbit IgG horseradish-peroxidase conjugate (1:5000, Goat anti-rabbit, Biorad) or anti-monkey IgG (1:400, Gamonkey, Pierce- Rockford. IL, USA). Light emission was measured after the addition of the Western lightning chemiluminescence reagent (NLE 101, Perkin Elmer) using an ImageQuant RT ECL (General Electric Healthcare).

### **Statistical analysis**

Samples were run in duplicate or triplicate. The results were expressed as the mean ± standard deviation. Comparisons between the two groups were analyzed by the nonparametric Mann–Whitney test. The null hypothesis was rejected when P values were < 0.05.

## **Results**

### **Intravenous delivery of rAAV5 under immunosupression is safe**

The IS regimen applied to the numbered and colour-coded macaques as described in Figure 1A, maintained almost undetectable peripheral blood CD19^+^ B-lymphocytes for the duration of the protocol as an effect of Rituximab (Additional file 1: Figure 1A). The intensive IS regime reduced the absolute numbers of CD4^+^ and CD8^+^ T cells in peripheral blood (Additional file 1: Figure 1B-C) as a direct effect of ATG administration immediately before each rAAV infusion. Daily administration of MMF and FK506 for 12 weeks was used to functionally repress the remaining T and B lymphocytes. Studies by multicolour flow cytometry in peripheral blood indicate that the immunosuppressive drugs used did not selectively change the percentage of CD4^+^FOXP3^+^ regulatory T cells (Tregs) compared with effector T cells (data not shown).

It is of note that the overall treatment did not cause worrying liver transaminase increases in serum and that blood counts including platelets were not altered in these non-human primates (Additional file 2: Figure 2). Moreover, upon rAAV vector administration there were only mild and transient increases in serum inflammation-denoting cytokines such as interleukin-6 (Additional file 3: Figure 3). Accordingly, the IS regime does not seem to let AAV vectors to cause more serious inflammatory reactions.

### **Immune responses to capsid antigens down-modulated by IS**

Direct intravenous injection of rAAV5 vector into a control naïve NHP001 macaque resulted in a robust antibody response (Figure 1B) and a moderate T-cell proliferative reaction against AAV5 capsid antigens (Figure 1C). The intensive IS regimen applied to NHP002 and NHP003 was able to restrain the humoral response (Figure 1B) and completely abolish the specific T cell responses to rAAV5 structural proteins as measured in a lymphocyte proliferation assay (Figure 1C). However, IS failed to attenuate the specific humoral response following withdrawal of the immunosuppressants and as a consequence the nAB titers rose about three-fold from the previously attenuated levels upon drug cessation (Figure 1B). Neutralizing antibodies detected in these macaques correlated with antibodies titers directed to adsorbed viral capsids in ELISA assays that, although showed lower sensitivity for detection, confirmed specificity (Additional file 4: Figure 4). Importantly, no antibodies directed against the human PBGD were detected by western blot assays at different time points through the study (Additional file 5: Figure 5).

### **IS retards serum clearance of proviral DNA**

It was observed that under pharmacological immunosuppression circulating proviral DNA persisted for much longer periods of time than in the control NHP001 macaque, with sustained proviral DNA levels up to fifteen weeks as detected by sequential QPCR. (Figure 1D). An *in vitro* transduction assay in PLC-PRF5 cells was performed with the serum from immunosuppressed animals at 2 and 7 weeks after rAAV5-*PBGD* administration. Lack of *in vitro* transduction of a sensitive cell line, as measured by specific QPCR in genomic DNA extracted from infected cells, suggested that the proviral DNA genomes in circulation did not represent infective viral particles. As a positive control for these experiments, rAAV5-*PBGD* vector readily caused productive transduction in parallel experiments at the same moi of 60 (data not shown).

### **IS fails to allow for repeated gene transfer with AAV vectors of the same serotype**

**Figure 2:**
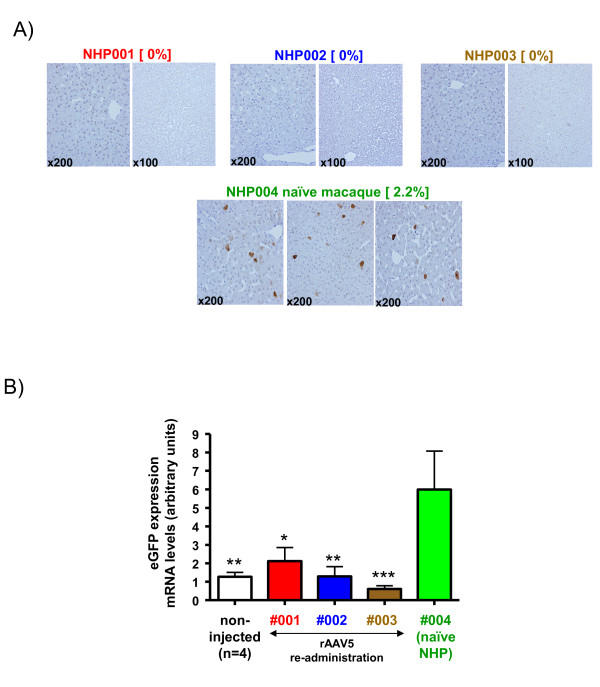
**Administration of second rAAV5 vector of the same serotype cannot achieve transgene expression even under immunosuppression of macaques with the five-drug regimen. A)** Representative microphotographs of liver sections from the necropsy material of the indicated animals upon immunostaining with an anti-eGFP polyclonal antibody. The positive control animal (NHP004) received only a rAAV-*egfp* injection as indicated in Figure 1. **B)** mRNA analysis by RT-PCR corresponding to specific *egfp* mRNA expression in liver of colour-coded animals. Data are expressed as median ± standard deviation of eight different lobes in each macaque. *, p < 0.05; **, p < 0.01; ***, p < 0.001 versus naïve non-human primate.

To study if re-administrations of rAAV5 were feasible in animals with the aforementioned antibody titers (Figure 1B), a rAAV5 vector encoding the *egfp* reporter gene was given to the macaques 23 weeks later. None of the macaques pre-exposed to the *PBGD*-encoding rAAV5 vector showed any liver expression of eGFP (Figure 2), while a control naïve macaque revealed measurable eGFP expression in the liver two weeks after vector administration (Figure 2A,B).

### **Transgene expression persistence in an immunosuppressed macaque**

**Figure 3:**
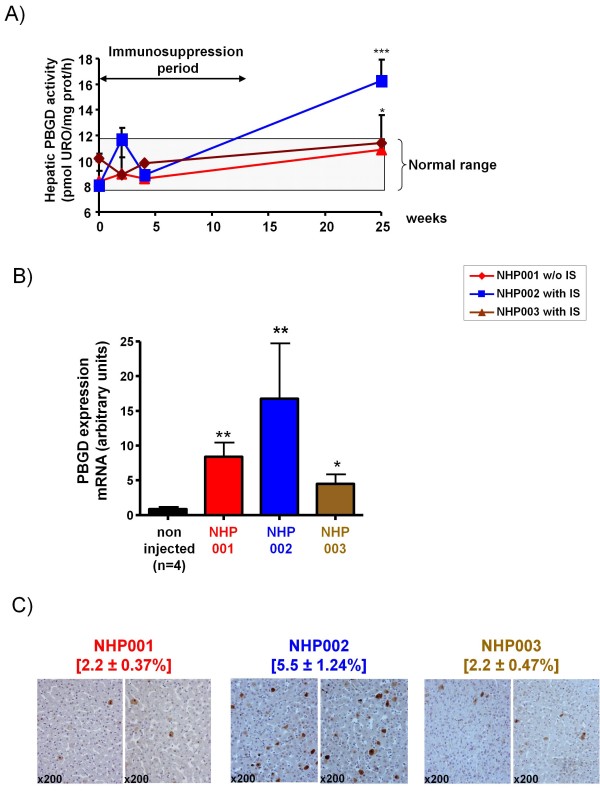
**Transient pharmacological immunosuppression fails to enhance rAAV5-mediated gene transfer to the liver in macaques. A)** Sequential follow-up of PBGD activity in homogenates from liver samples taken at the indicated time points. Normal range was defined as means ± 2 standard deviation of a separate group of four non-injected female macaques. **B)** Analyses of human *PBGD* mRNA content by quantitative RT-PCR in liver homogenates from necropsies taken at sacrifice (week 25). **C)** Immunohistochemistry detection of hepatocytes overexpressing PBGD on formalin-fixed and paraffin-embedded sections from liver biopsies obtained from the indicated animals at sacrifice. Data corresponding to necropsy samples (week 25) are expressed as median ± standard deviation of eight different lobes in each macaque.

A second aim of our study was to analyze the ability of the transient IS regime to prolong transgene expression following the first injection of rAAV5-*PBGD*. Successful liver gene transfer following vector injection was confirmed by quantitative measurements of vector DNA in liver biopsies performed 15 days after the administration of the rAAV5-*PBGD* vector. To avoid “hit or miss” biopsy problems due to presumably non homogeneous expression of the transgene in the target organ, we took two needle biopsies from the right lobe and two more samples from the left lobe. No difference in vector genome copies in liver biopsies were observed between macaques with or without IS (data not shown). However, hepatic PBGD activity was not found to be increased in biopsies from any of the macaques during the first month after administration of rAAV5-*PBGD* (Figure 3A). Of interest, the liver of NHP002 showed increased transgene expression as late as 25 weeks after vector administration, once having been free from IS for 13 weeks (Figure 3A). The macaque NHP003 which received the second vector administration again under IS did not show detectable increases in PBGD enzymatic activity (Figure 3B). These data were confirmed in liver samples taken upon necropsy at week 25 both at the mRNA level (Figure 3B) and upon immunohistochemical staining (Figure 3C). A possible interpretation to the detection of long-lasting transgene expression in NHP002 following IS cessation is that one or some of the IS agents could be interfering with transgene expression as conferred by rAAV5 vectors, although factors of individual variability could also be behind these results. The possibility of drug interference with gene expression was subsequently addressed in experiments performed in mice.

### **MMF represses AAV-encoded transgene expression in the liver of mice**

**Figure 4:**
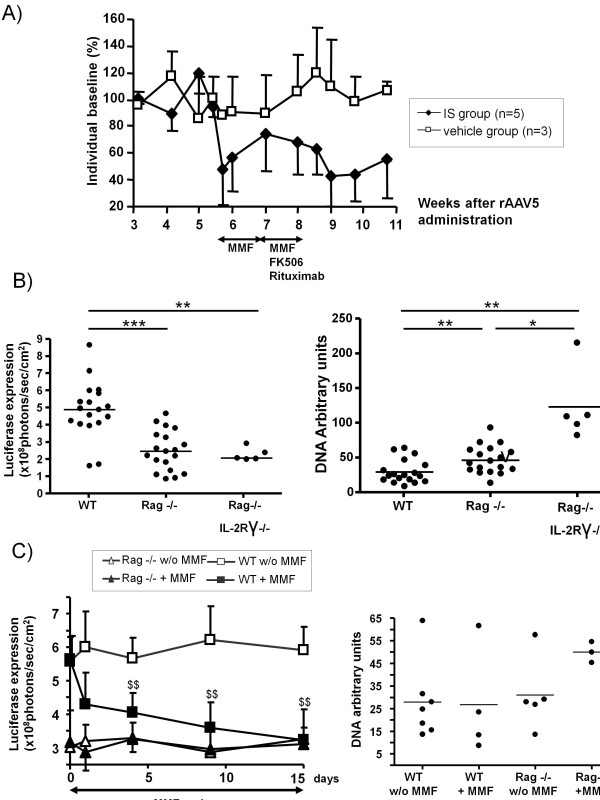
**Mycophenolate mofetil (MMF) interference with transgene expression in mice. A)** Sequential follow-up by bioluminescence of hepatic luciferase expression following an *i.v.* dose of 5x10^12^ genome copies of rAAV5-luciferase/kg in BALB/c mice with or without immunosuppressants at the indicated time points. **B)** The left graph represents luciferase expression measured as bioluminescence in the livers of Rag−/−, Rag−/−IL-2Rγ−/− and wild type syngeneic BALB/c mice 3 weeks after vector administration. The right graph shows proviral DNA content assessed by quantitative PCR in the liver of the same animals. **C)** The left graph represents sequential follow-up of hepatic luciferase expression following rAAV5-luciferase/kg injection in BALB/c mice and Rag−/− mice. Four weeks following vector administration daily MMF was given for 15 days to the indicated groups. The right graph shows vector DNA quantification in the liver when mice were sacrificed three days after MMF cessation (45 days after vector infusion). ++, P < 0.01 versus wild type mice without immunosuppression.

Administration of MMF to groups of BALB/c mice transduced with rAAV5 encoding *luciferase* led to lower transgene expression in terms of photon emission upon bioluminescence analysis (Figure 4A). The addition of FK506 and Rituximab to the treatment regimen did not significantly further reduce transgene expression (Figure 4A), thus indicating that MMF was the main factor decreasing expression of the transgene.

MMF may have effects on target immune system cells as well as immune-independent off-target effects. To ascertain whether the reduction of transgene expression was related to the existence of an adaptive immune system, we compared rAAV5 expression in wild type and Rag^−/−^ BALB/c mice, which are deficient in T and B lymphocytes. Expression of the luciferase transgene activity was approximately twice more intense in the wild type animals than in the Rag^−/−^ mice (Figure 4B left). Nonetheless, the amount of proviral DNA in the liver was similar, or even greater in immunodeficient than immunocompetent mice (Figure 4B right), indicating that it is not a matter of liver gene transfer but of transgene expression. Similar data were observed with independently bred Rag^−/−^ IL-2Rγ^−/−^ double knock-out mice that are also deficient in NK lymphocytes in addition to the lack of mature T and B cells (Figure 4B).

To define if MMF was exerting its effects through the cellular immune system, we performed comparative experiments in Rag^−/−^ and wild type mice, with or without treatment with MMF. Importantly, MMF was administered four weeks after injection of our single stranded AAV5 vector in order to avoid its interference effect on second-strand DNA synthesis [19,20]. MMF only decreased luciferase expression over baseline levels in the case of immunocompetent animals (Figure 4C left). Such a decrease in expression of luciferase despite similar levels of vector DNA (Figure 4C right), again indicates that MMF decreases transgene expression, but interestingly only when an adaptive immune system is present in the animal.

## **Discussion**

Long lasting gene expression attained by rAAV-mediated gene transfer to the liver can be curative in a number of life-threatening metabolic diseases [2,4]. The genome of the rAAV vector persists predominantly in episomal forms, thereby increasing safety by reducing the risk of insertional mutagenesis. However, this feature may favour loss of genomes and transgene expression over time due to hepatocyte turn-over. Drug immunosuppressants create unresponsiveness to the viral capsid proteins and may provide a strategy to allow repetitive administration of the same vector.

In our animals, intravenous delivery of rAAV5 activates significant humoral responses to AAV capsids but not to the cytosolic transgene product, which in the case of PBGD is an endogenously expressed protein that is highly conserved (99.2 % identity) between humans and *macaca*. Most probably, PBGD transgene protein will not elicit immune responses in a clinical trial for acute intermittent porphyria because the normal allele of PBGD produced in all cells from these patients should have induced immunological tolerance to the identical pbgd transgene-encoded protein.

Notably, this is the first report performing rAAV administration under B-cell depleting treatment with rituximab. The humoral response against the AAV capsid was reduced in the two macaques during the three month period of intensive IS regimen when compared to the control macaque. Reduction was not complete denoting residual B cells and T-cell help for such a strong immunogen. Once the IS protocol was halted, the nAB levels reached the values obtained in the control macaque that did not receive IS. These data strongly indicate that upon IS cessation, the persistence of capsid antigens associated in some form with long-lasting viral DNA permits a boost in the generation of anti-capsid antibodies.

In previous work from other laboratories performed in rhesus macaques, in only one animal of the three treated daily with MMF and tacrolimus for 6 weeks did the levels of anti-AAV8 IgG antibodies increase after IS therapy was stopped [12]. In another study, daily treatment with MMF and sirolimus for 10 weeks reduced humoral response against AAV2 capsid in rhesus macaques [10]. The anti-AAV2 nAB titer declined by week 13 and no rebound peak in the titer was observed following discontinuation of the IS regimen. These data suggest that the inclusion of rituximab in our regimen resulted in antigen persistence that becomes immunogenic following IS cessation. This can be interpreted in the sense that B cells and their production of antibodies are important for AAV antigen clearance. Hence, immunosuppressive regimens for AAV should permit clearance of vector antigens, while suppressing neutralizing humoral immunity. A recent report in mice suggests the use of non-depleting anti-CD4 monoclonal antibodies combined with cyclosporine-A induced immunological tolerance mediated by regulatory T cells instead of immunosuppression [21]. Induction of antigen-specific tolerance to the AAV vector, as opposed to general immunosuppression, would be the best solution to a difficult problem and future research should focus on this matter.

Data from a clinical trial of hemophilia B gene therapy with a rAAV2 vector [2,8,9] show that an increase in liver enzyme levels was associated with the generation of cytotoxic CD8 + T cell response specific for the rAAV2 capsid protein [9]. The finding that the cytotoxic T lymphocyte response eliminated gene expression in transduced cells was not observed in the clinical trial with rAAV8 trial where a course of glucocorticoid therapy was given [16]. It could be that the more rapid uncoating of rAAV8 than of rAAV2 capsid proteins from viral particles [22,23] allows the rAAV8 capsid proteins to be degraded by transduced cells before the immune system can seek and destroy them. Several studies have suggested that rate of uncoating of vector genomes is lower with rAAV2 than with serotypes rAAV5, rAAV6 and rAAV8 [18,23]. These results raise the possibility that serotypes different from rAAV2 behave less efficiently to develop T-cell mediated adaptive immune responses.

In the present report, the control NHP showed a moderate T-cell proliferative reaction against AAV5 capsid antigens. This specific T cell response was completely abolished in the two animals that received immunosuppression. It is of interest that transgene expression was detectable six months after vector infusion in one of our macaques that received IS circa the first administration of vector. However, it had not been detectable in liver biopsies taken from the same animal two and four weeks after vector infusion while the animal was still under IS. This finding raises the issue of a potentially deleterious side effect of IS on transgene expression. Recent work [19] demonstrated that MMF inhibits the synthesis of the complementary DNA strand following entry of the rAAV into the target cells. In addition to this mechanism, we have experimentally documented in this study that MMF impairs transgene expression even when the transcriptionally active form of the provirus is already in place. The MMF effect required a functional adaptive immune system since this effect was not observed in Rag^−/−^ mice. Such an effect of MMF constitutes an unexpected result given that MMF is well studied as a repressor of immune functions. Paradoxical actions of MMF as a repressor of immune functions and in controlling hepatitis C virus (HCV) [24]and hepatitis B virus [25] have been reported *in vitro*. The mechanism of action on anti-HCV replication is still unknown but reported to be independent of cell proliferation and guanosine depletion [26].

Regardless of the mechanism, this pharmacological unexpected silencing effect of MMF is of particular relevance when considering the use of MMF to improve the outcome of rAAV-mediated gene therapies. Our findings are in line with reference 24 and represent a warning for the design of future clinical trials.

## **Conclusions**

IS controls the cellular immune response to AAV and one out of two macaques showed liver trangene expression 6 months after vector administration. However, our results indicate that rAAV5-liver mediated transgene expression in *Macaca fascicularis* poses immune-mediated obstacles that are unsatisfactorily overcome by the potent immunosuppressants used in combination. The reasons include: (i) incomplete restraint of humoral immune responses to viral capsids, (ii) an intriguing MMF-dependent drug-mediated interference with liver transgene expression that takes place via a so far elusive mechanism exerted with a requirement for a functional adaptive immune system.

## **Abbreviation**

rAAV, Adeno-associated vectors; PBGD, Porphobilinogen deaminase; MMF, Mycophenolate mofetil; nABs, neutralizing antibodies; AIP, acute intermittent porphyria; FK506, Tacrolimus; ATG, Rabbit antithymocytic globulin; IS, Immunosuppressive; eGFP, Enhanced green fluorescence protein; EalbAAT promoter, Human α-1-antitrypsin promoter with regulatory sequences from the human albumin enhancer; QPCR, Quantitative polymerase chain reaction; NHP, Non-human primates; S.I., Stimulation index; cpm, Counts per minute.

## **Competing interests**

Stuart G. Beattie and Harald Petry are employees of Amsterdam Molecular Therapeutics. There are not financial competing interests to declare in relation to this manuscript.

## **Authors’ contributions**

IgM, AF conceived of the study. CU, SHS, AB, JP, IgM, AF participated in the design of the study and performed the statistical analysis. AB, AF carried out the samples extraction. CU, AS, ItM, AF carried out the molecular and biochemical studies. SHS, UM, CA carried out the cellular immunity response. CU, ItM carried out the neutralizing antibodies analysis and participated in immunoassays. CU, SGB, AF, IgM drafted the manuscript. REdeS, HP, JP helped to draft the manuscript. All authors read and approved the final manuscript.

## **Authors’ information**

The authors form a translational research team on the borders between gene therapy and immunology/immunotherapy. Our efforts are centred on immunotherapy of cancer and liver gene transfer with therapeutic purposes. The team combines expertise in modulation of the immune system and gene therapy for porphyrias. Particular emphasis is placed on a translational point of view that is reflected in clinical trials including one ongoing trial for gene therapy correction of acute intermittent porphyria.

## Supplementary Material

Additional file 1 **Figure S1.Follow-up of the absolute numbers of A) CD19**^**+**^**B-lymphocytes, B) CD4**^**+**^**T-cells and C) CD8**^**+**^ T-cells in the peripheral blood of the indicated colour-coded macaques measured by flow cytometry. Of note immunosuppressed animals on day 0 had already been treated with rituximab since day −9. Click here for file

Additional file 2 **Figure S2.** Follow-up of platelet counts, Aspartate Aminotransferase (AST) and Alanine Aminotransferase (ALT) in the sera from non-human primates after the second rAAV serotype 5 administration. Click here for file

Additional file 3 **Figure S3.** Serum interleukin-6 concentrations after A) the first and B) second AAV5 administrations to the indicated NHP. Click here for file

Additional file 4 **Figure S4.** Anti AAV5-capside antibody titer follows up by ELISA in sequential serum samples of the indicated macaques. Click here for file

Additional file 5 **Figure S5.** Lack of humoral response to the transgene protein Immunoblot analysis of hPBGD recombinant protein with serum from macaques injected with rAAV5-pbgd (1:3 dilution). An anti-PBGD specific polyclonal antibody was used as a positive control. Click here for file
