# Supplementary material for: Transient and intensive pharmacological immunosuppression fails to improve AAV-based liver gene transfer in non-human primates
Source: J Transl Med. 2012 Jun 15;10:122. doi: 10.1186/1479-5876-10-122 (PMC3412719; doi:10.1186/1479-5876-10-122)
Supplement: Additional file 1 — Figure S1.Follow-up of the absolute numbers of A) CD19+B-lymphocytes, B) CD4+T-cells and C) CD8+ T-cells in the peripheral blood of the indicated colour-coded macaques measured by flow cytometry. Of note immunosuppressed animals on day 0 had already been treated with rituximab since day −9. [file 1479-5876-10-122-S1.ppt]

## Slide 1
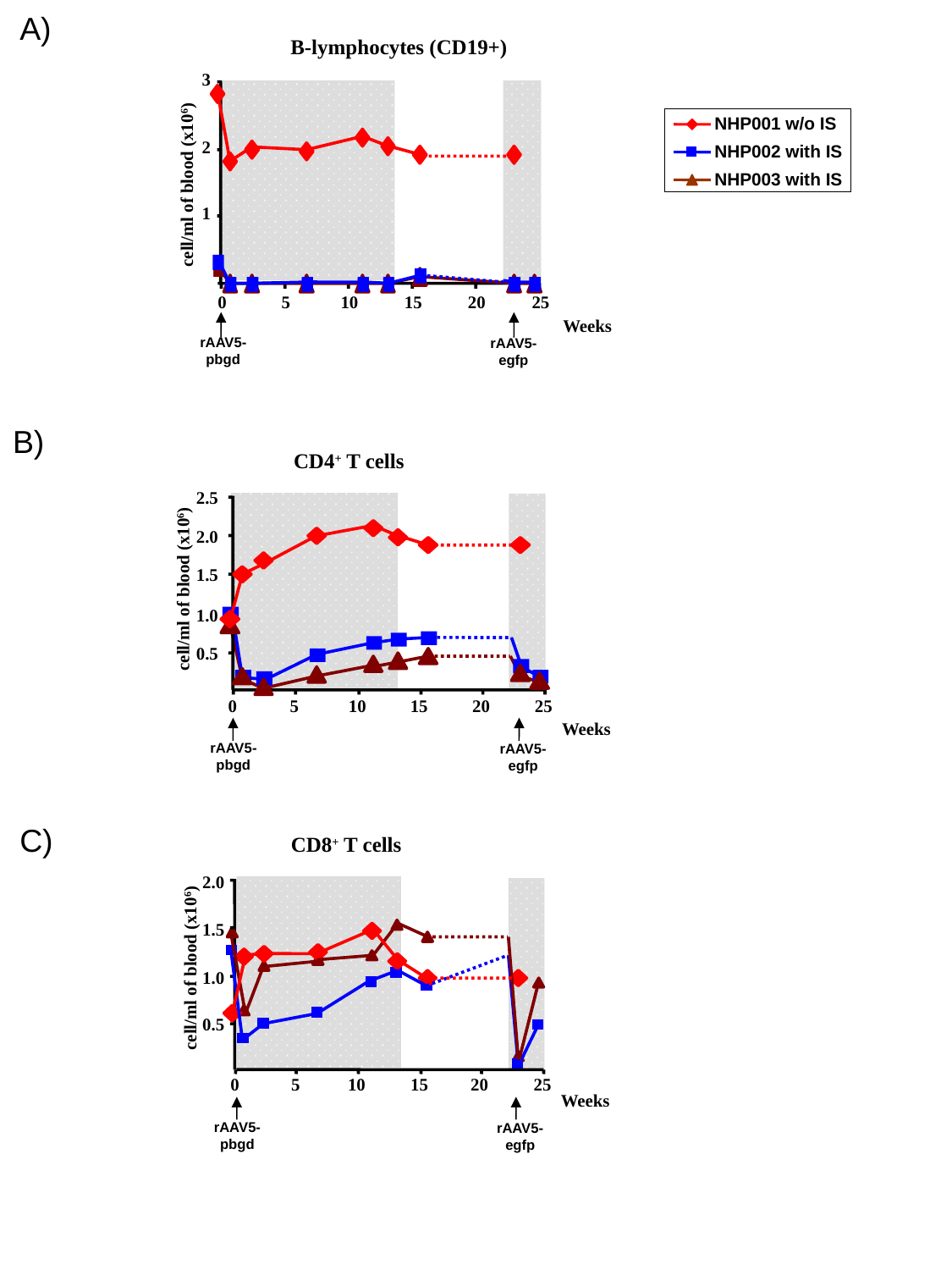

A)
B-lymphocytes (CD19+)
cell/ml of blood (x106)
3
NHP001 w/o IS
NHP002 with IS
NHP003 with IS
2
1
0
5
10
15
20
25
Weeks
rAAV5-
pbgd
rAAV5-
egfp
B)
CD4+ T cells
2.5
2.0
1.5
cell/ml of blood (x106)
1.0
0.5
0
5
10
15
20
25
rAAV5-
pbgd
rAAV5-
egfp
Weeks
C)
CD8+ T cells
2.0
1.5
cell/ml of blood (x106)
1.0
0.5
0
5
10
15
20
25
Weeks
rAAV5-
pbgd
rAAV5-
egfp
