# Supplementary figures and images for: Transient and intensive pharmacological immunosuppression fails to improve AAV-based liver gene transfer in non-human primates
Source: J Transl Med. 2012 Jun 15;10:122. doi: 10.1186/1479-5876-10-122 (PMC3412719; doi:10.1186/1479-5876-10-122)

## Slide 1
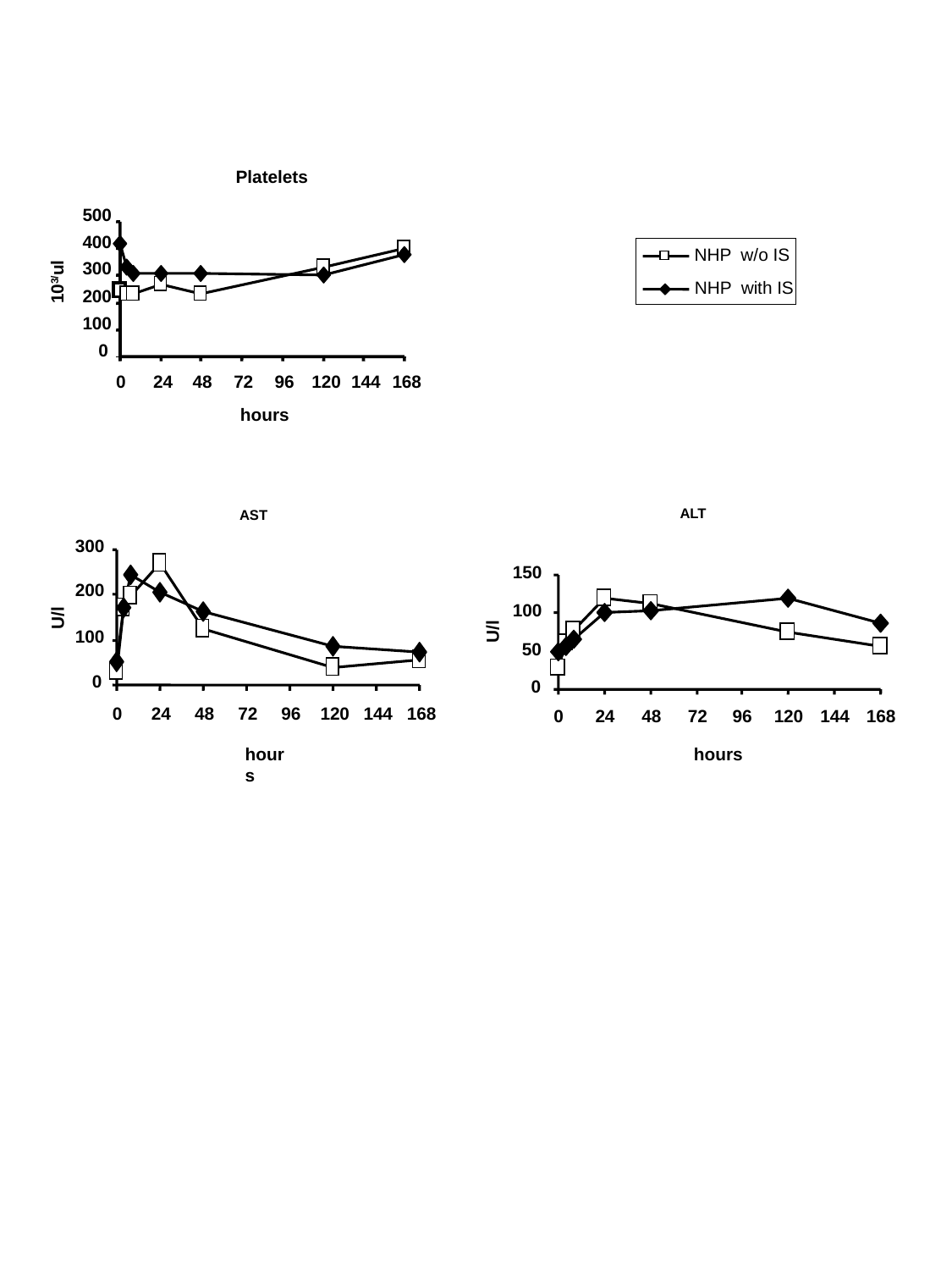

Platelets
500
400
300
103/ul
200
100
0
0
24
48
72
96
120
144
168
hours
NHP w/o IS
NHP with IS
ALT
AST
300
200
U/l
100
0
0
24
48
72
96
120
144
168
hours
150
100
U/l
50
0
0
24
48
72
96
120
144
168
hours

Supplement: Additional file 2 — Figure S2. Follow-up of platelet counts, Aspartate Aminotransferase (AST) and Alanine Aminotransferase (ALT) in the sera from non-human primates after the second rAAV serotype 5 administration. [file 1479-5876-10-122-S2.ppt]

## Slide 1
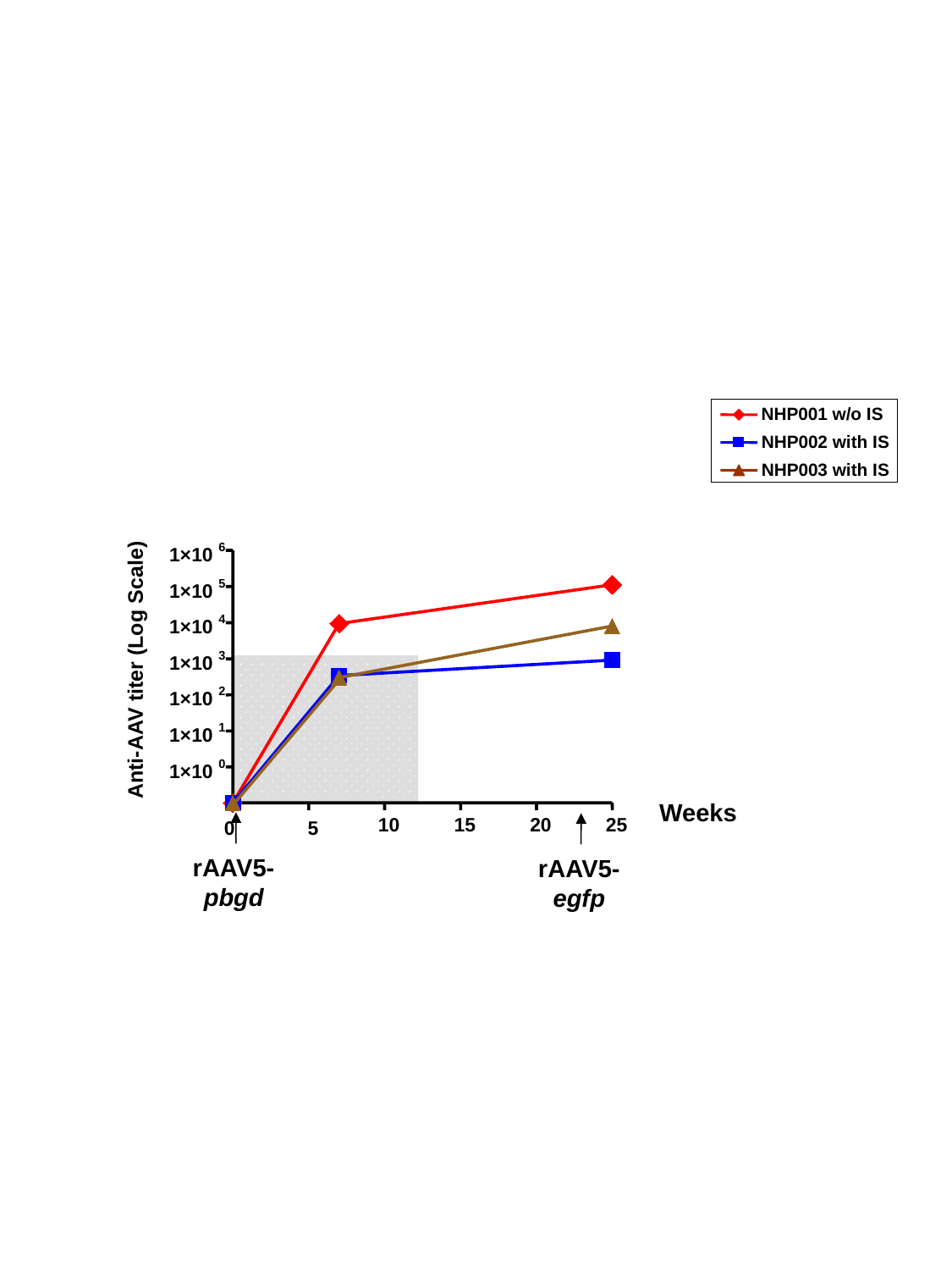

NHP001 w/o IS
NHP002 with IS
NHP003 with IS
6
1×10
5
1×10
4
1×10
3
1×10
Anti-AAV titer (Log Scale)
2
1×10
1
1×10
0
1×10
10
15
20
25
0
5
Weeks
rAAV5-
pbgd
rAAV5-
egfp

Supplement: Additional file 4 — Figure S4. Anti AAV5-capside antibody titer follows up by ELISA in sequential serum samples of the indicated macaques. [file 1479-5876-10-122-S4.ppt]

## Slide 1
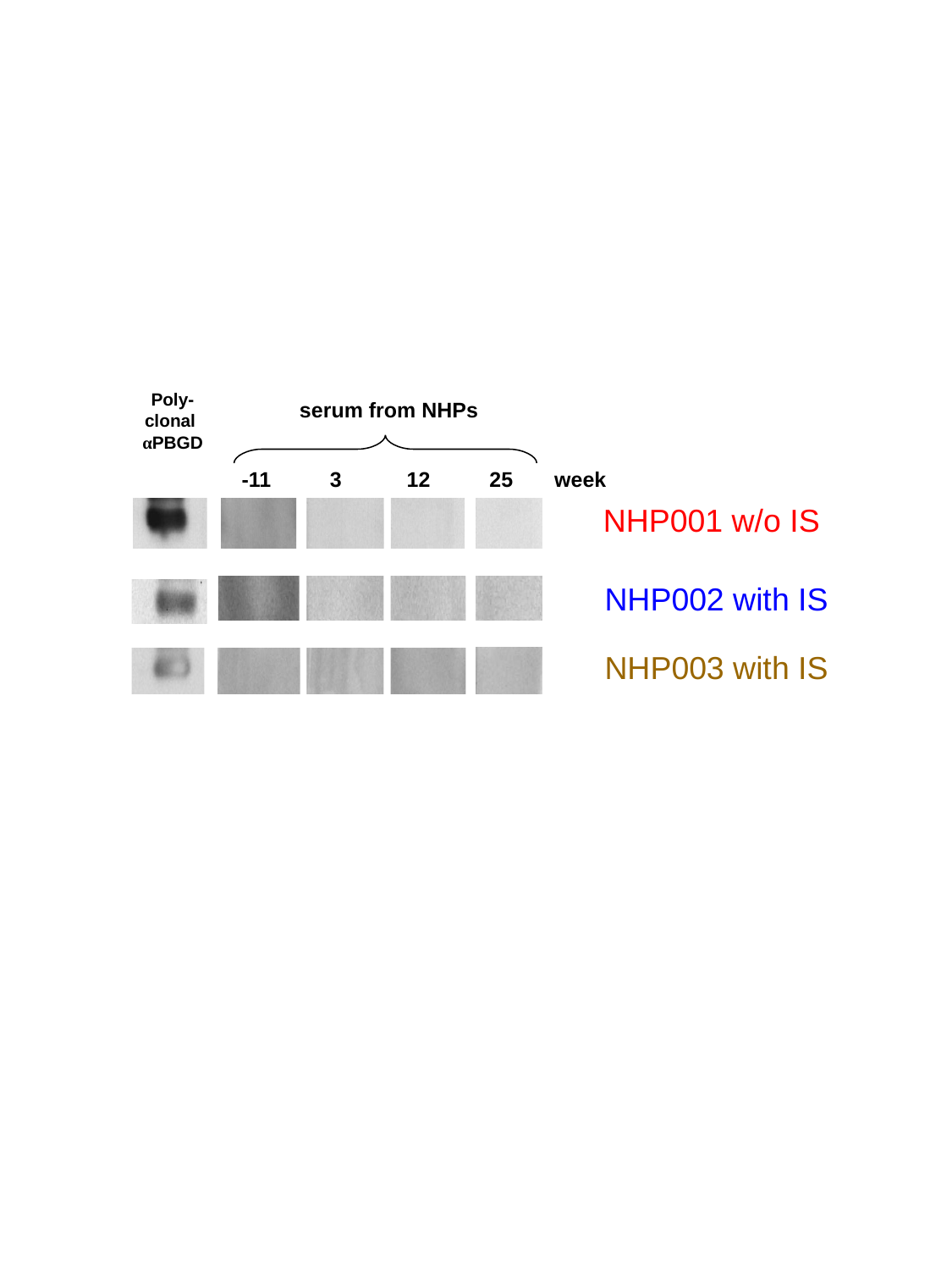

Poly-
clonal
αPBGD
serum from NHPs
-11 3 12 25 week
NHP001 w/o IS
NHP002 with IS
NHP003 with IS

Supplement: Additional file 5 — Figure S5. Lack of humoral response to the transgene protein Immunoblot analysis of hPBGD recombinant protein with serum from macaques injected with rAAV5-pbgd (1:3 dilution). An anti-PBGD specific polyclonal antibody was used as a positive control. [file 1479-5876-10-122-S5.ppt]
