# Supplementary material for: Transient and intensive pharmacological immunosuppression fails to improve AAV-based liver gene transfer in non-human primates
Source: J Transl Med. 2012 Jun 15;10:122. doi: 10.1186/1479-5876-10-122 (PMC3412719; doi:10.1186/1479-5876-10-122)
Supplement: Additional file 3 — Figure S3. Serum interleukin-6 concentrations after A) the first and B) second AAV5 administrations to the indicated NHP. [file 1479-5876-10-122-S3.ppt]

## Slide 1
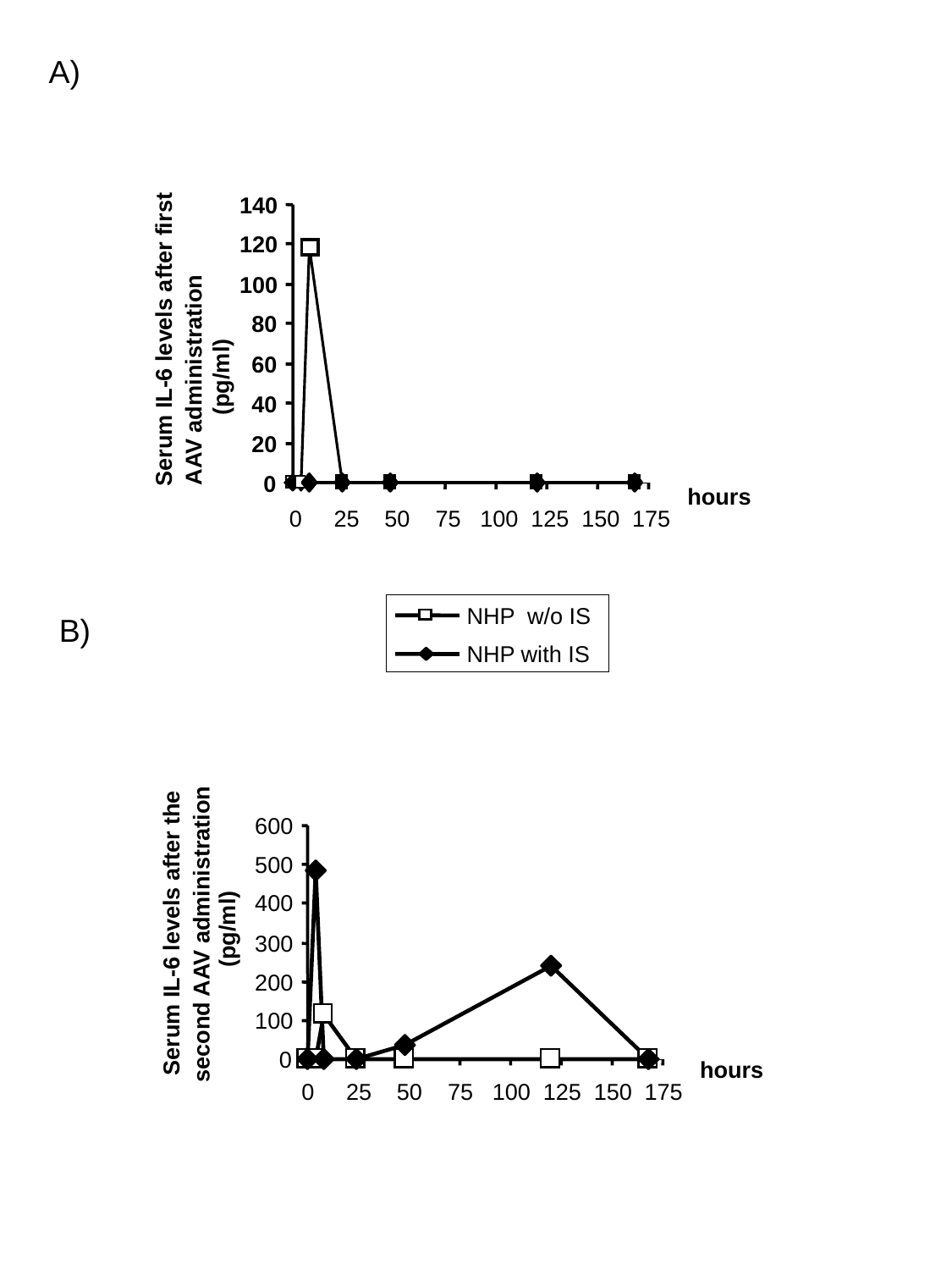

A)
140
120
100
80
Serum IL-6 levels after first
AAV administration
(pg/ml)
60
40
20
0
hours
0
25
50
75
100
125
150
175
NHP w/o IS
NHP with IS
B)
600
500
400
(pg/ml)
Serum IL-6 levels after the
second AAV administration
300
200
100
0
hours
0
25
50
75
100
125
150
175
